# Prehabilitation Reduces Occurrence of Anastomotic Leaks After Esophagectomy—A Retrospective Cohort Analysis and Meta-analysis

**DOI:** 10.1007/s12029-025-01213-z

**Published:** 2025-06-11

**Authors:** Alissa Jell, Alexandra Dusi, Marcus Feith, Jeannine Bachmann, Dirk Wilhelm, Marc Martignoni, Ekin Ihsan Demir, Helmut Friess, Stephan Schorn

**Affiliations:** 1 Department of Surgery, School of Medicine and Health, TUM Klinikum Rechts Der Isar, Technical University of Munich, Ismaninger Str. 22, 81675 Munich, Germany; 2 TUM Klinikum Rechts Der Isar, Medical Controlling Technical University of Munich, Munich, Germany

**Keywords:** Esophagectomy, Anastomotic leakage, Anastomotic insufficiency, Prehabilitation, Systematic review, Meta-analysis

## Abstract

**Background:**

Esophageal anastomotic leaks (EAL) after esophagectomy strongly increase postoperative mortality and morbidity. Identifying, addressing, and improving risk factors are pivotal. In this article, we conducted a systematic review with meta-analysis, comparing findings with our 13-year experience in a German high-volume esophageal surgery center.

**Methods:**

Databases of Pubmed, Scopus, and Cochrane were systematically screened for publications prior to 2025, and all patients undergoing esophageal resection surgery from 2010 to 2022 were analyzed for EAL occurrence, incorporating the review data into our analysis.

**Results:**

Among 14,163 studies screened, 202 were included, with 123 studies providing sufficient information on risk factors’ impact on EAL. Our patient register revealed 144 out of 787 with EAL. Cardiopulmonary factors such as hypertension (RR 1.44; *p* = 0.0004), coronary artery disease (RR 1.28; *p* = 0.0004), heart insufficiency (RR 1.56; *p* = 0.05), peripheral artery disease (RR 1.65; *p* = 0.0009), pulmonary disease (RR 1.5; *p* = 0.01), COPD (RR 1.39; *p* = 0.13), renal insufficiency (RR 1.61; *p* = 0.03), diabetes mellitus (RR 1.51; *p* < 0.00001), obesity (BMI > 25; RR 1.31; *p* = 0.009; BMI > 30; RR 1.49; *p* = 0.006), smoking (former smoker: RR 1.54; *p* < 0.0001; active smoker: RR 1.25; *p* < 0.0001), and frequent alcohol intake (RR 1.7; *p* = 0.003) were all associated with an increased risk of EAL following esophagectomy. We show that preoperative management targeting these risk factors result in a significant reduction of EAL.

**Conclusion:**

Our extensive review underscores the critical role of cardiovascular, pulmonary, and renal conditions in EAL development, emphasizing the importance of prehabilitation to mitigate risks associated with EAL after esophagectomy.

## Introduction

Despite advancements in surgical techniques and postoperative intensive care, esophageal anastomotic leaks (EAL) remain as one of the most devastating postoperative complications following esophagectomy. Recent studies indicate the incidence of EAL after esophagectomy still ranges between 8 and 19% [1, 2]. The profound impact of EAL becomes evident when examining both short- and long-term outcomes. EAL is associated with worsened short-term parameters, including mortality, readmission rates, early cancer recurrence, overall survival, hospital stay duration, costs, resource utilization, and quality of life. Regarding in-hospital mortality, Whooley et al. [3] reported a threefold increase in patients with EAL to those without EAL. Similarly, Rutegard et al. [4] showed that the 90-day survival rate dropped by more than half in patients with EAL (18.9% vs. 6.2%). EAL is strongly associated with an increase in overall postoperative complications reaching 100% in severe cases (*n* = 208/208) compared to 51.9% in the general cohort (*n* = 1266/2439) [5]. Hospital stays were prolonged with 27 days compared to 15 days for those without EAL [3]. Kassin et al. [6] reported increased readmission rates among patients with postoperative complications after esophagectomy compared to those without (odds ratio/OR 4.20; 95% confidence interval (CI) 2.89–6.13). Strikingly, negative effects of postoperative complications extend beyond the immediate postoperative period, with long-term impacts on health-related quality of life. Derogar et al. [7] assessed the quality of life in 141 patients 5 years after esophagectomy of whom 46 had major postoperative complications such as EAL, postoperative bleeding, and sepsis. Major postoperative complications were associated with increased fatigue and eating restrictions.

Given this substantial burden on patient outcomes, characteristics of stratifying patients into high- and low-risk groups are crucial. Several studies have explored the impact of patient characteristics on the occurrence of postoperative adverse events and anastomotic leaks. Medical history of hypertension, coronary disease, vascular disease, diabetes mellitus, renal insufficiency, high body mass index (BMI), respiratory disease and tobacco use, parameters as forced expiratory volume in one second percent (FEV1%), serum creatinine, hypoproteinemia, degree of differentiation of malignant tumours, a prolonged procedural duration (> 5 h), and the type of procedure have already been identified as unfavorable predictors of postoperative complications [7–10]. This knowledge led to the implementation of tools for risk stratification in 2020 [11] and mortality in 2021 [12]. However, the clinical relevance of the risk factors and their scorings vary depending on study design, patient population, and do not account for long-term outcomes or the impact on prehabilitation.

## Methods

### Study Design

All patients undergoing esophagectomy in the period January 2010 to December 2022 at our Department for Surgery, a German very high-volume esophageal center and University hospital, were screened for postoperative EAL. To enhance screening accuracy, all patients undergo postoperative endoscopy on day 5 in our center [13]. If EAL was found positive, those patients were retrospectively worked up for patient specific data (age, sex, BMI), their past medical history (PMH: diabetes mellitus, hypertension, coronary disease, heart and renal insufficiency, pulmonary disease, steroid treatment, ASA classification, and history of previous malignancy), drug history (tobacco and alcohol use), and cancer specifications (TNM classification, neoadjuvant chemotherapy, and radiation).

According to the recommendations of the Cochrane Network, the reporting guideline of The Preferred Reporting Items for Systematic review and Meta-Analysis (PRISMA) [14] was considered for this systematic review with meta-analysis. To get an overview of the literature, databases of Pubmed, Scopus, and Cochrane were screened for the search terms “esophagectomy,” anastomotic “insufficiency,” “anastomotic leak,” “anastomotic leaks,” “anastomotic leakage,” and “anastomotic leakages.” All data published prior to January 2025 were screened. All studies published in an English language peer-reviewed journal were eligible for the review. The entire systematic review and meta-analysis protocol was registered at PROSPERO (CRD42017060841).

#### Inclusion and Exclusion Criteria

After removing duplicates, the literature was screened for inclusion and exclusion criteria and the database for the systematic review was build. All studies were presented to two out of three independent reviewers (AD, AJ, SS), and disagreements were shown to a third reviewer (EID, SS). For the entire screening-process, the following inclusion and exclusion criteria were used:

##### Surgical techniques

All studies were carefully screened for the surgical technique used to ensure that the data were as homogeneous as possible. For this purpose, two reviewers independently screened the “methods” part of each manuscript. Only papers with abdomino-thoracic, abdomino-cervical esophagectomy with gastric pull-up, or transhiatal esophagectomy were included in the systematic review. Papers with other operation techniques including colon interposition were consequently excluded as these techniques demand other anatomic and vascular challenges.

##### Histology

Although most studies included in our systematic review enrolled patients with malignant diseases, no restrictions were set for diseases requiring esophagectomy. However, pediatric surgery was excluded as these patients harbor a different risk profile for postoperative complications compared to adult patients.

##### Study design

Since the primary focus of the study was the occurrence of EAL after esophagectomy, all studies were included regardless of their design or length of follow-up as clinically relevant EAL would be expected to occur during the hospital stay the surgery was performed. Congress articles, reviews, systematic reviews, and all other studies with no primary data were excluded but were screened for supportive and additional source data.

#### Data Extraction and Statistical Analysis

After completion of the systematic review database, risk factors and the development of EAL were extracted to calculate risk ratios (RR), their corresponding 95% confidence interval (CI), and *p*-values. For continuous variables, means and their corresponding standard deviation or *p*-values were extracted and MD with corresponding standard deviations (SD) was calculated in the meta-analysis.

To perform the meta-analysis, the Review Manager Software (RevMan, Version 5.4, Copenhagen, The Nordic Cochrane Centre, The Cochrane Collaboration, 2012) was chosen. Heterogeneity was assessed using the inconsistency statistic (*I*^2^). The severity of heterogeneity was categorized as follows: no serious degrees if *I*^2^ was zero or less than 50%, and serious if *I*^2^ exceeded 50%.

The Mantel–Haenszel method for random effects was used for all meta-analysis to pool RR. All RRs were expressed with their associated 95% CI and *p*-values. In cases involving continuous variables, the MD was calculated using the inverse variance model for random effects and expressed with their corresponding SD and *p*-value. A two-sided *p*-value was calculated for each meta-analysis considering a significance level of *α* = 0.05.

#### Quality Assessment and Risk of Publication Bias

After establishing the review database, all full-text articles were screened according to “The Strengthening the Reporting of Observational Studies in Epidemiology” guidelines (STROBE16 criteria) [15]. Therefore, each study was evaluated for those STROBE criteria and percentages were calculated reaching from 0 indicating the worst study design to 100 for a perfectly designed study.

## Results

**Fig. 1:**
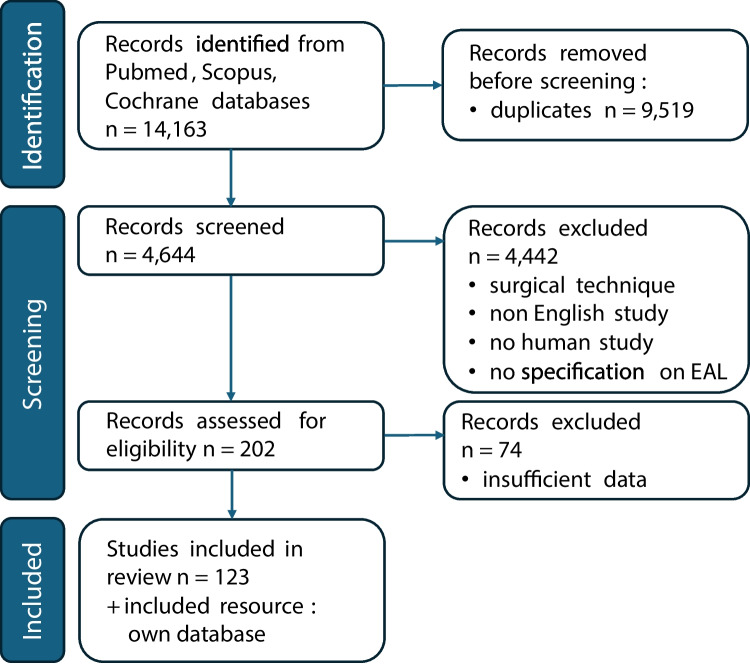
The PRISMA flowchart

A total of 14,163 studies were identified by screening PubMed, Scopus, and Cochrane central databases illustrated by the PRISMA flowchart (Fig. 1). Of these, 202 studies remained in our systematic review database after screening studies according to predefined inclusion and exclusion criteria, whereas only 123 studies provided sufficient data to be pooled into the different meta-analyses. Of the included papers, six were randomized controlled trials, 12 prospective, and 104 retrospective studies. Additionally, our analysis included our institution’s data, comprising all patients (*n* = 787) who underwent esophagectomy between 2010 and 2022 of which 144 developed postoperative EAL.

### Impact of Demographic Parameters on the Development of EAL

**Fig. 2:**
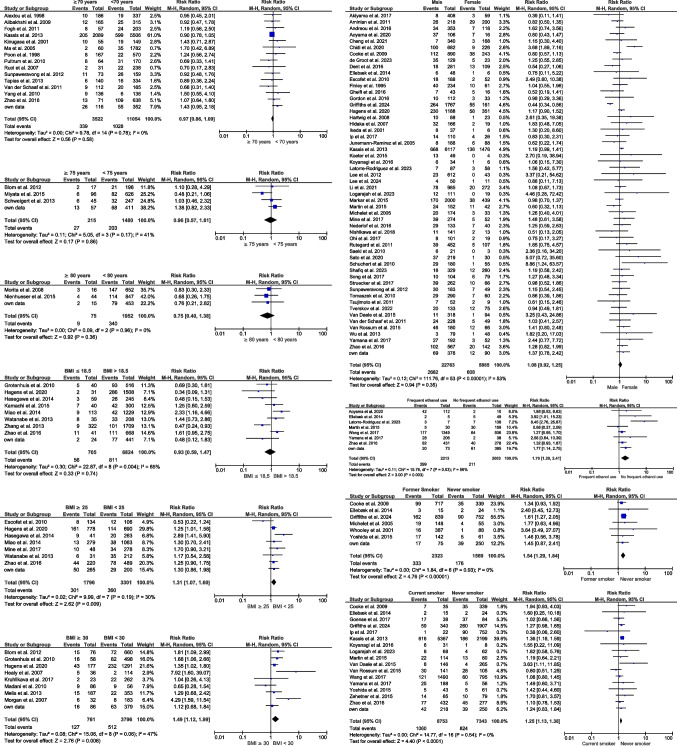
Impact of demographic parameters on the development of EAL

To assess the impact of patients’ age, 15 studies with 14,576 patients could be identified and pooled in the meta-analysis with a cut-off age of 70 years [10, 16–28], whereas there were four studies [29–31] with 1695 patients with a cut-off age of 75 years and three studies [32, 33] with 2027 patients with a cut-off age of 80 years. Importantly, none of these meta-analyses could detect any clinically relevant impact of the age on the occurrence of EAL (70 years; RR 0.97; [95% CI 0.86, 1.09; *p* = 0.58]; 75 years: RR 0.96; [95% CI 0.57–1.61; *p* = 0.86]; 80 years: RR 0.75; [95% CI 0.4–1.38; *p* = 0.36]) (Fig. 2) while own data showed mean ages of patients with EAL to be 2.67 years older than their controls (95% CI 0.258–5.125; *p* = 0.03).

Esophageal cancer affects more male than female patients. In 54 studies [1, 2, 4, 5, 8, 10, 12, 24, 26, 28, 34–77] with a total of 28,628 patients (79.5% male) (RR 1.08; 95% CI 0.92–1.25: *p* = 0.35), no clinically relevant effect could be seen on the impact of sex on EAL (Fig. 2).

To assess the impact of bodyweight on the occurrence of EAL, meta-analyses were performed divided into four groups: BMI below 18.5 kg/m^2^ [1, 9, 28, 78–82], normal BMI, BMI above 25 kg/m^2^ [1, 9, 28, 43, 58, 80, 82], and BMI above 30 kg/m^2^ [1, 29, 78, 83–87]. Those meta-analyses could show a significant increase of EAL in obese patients increasing with the BMI (BMI > 25 kg/m^2^: RR 1.31; 95% CI 1.07–1.60; *p* = 0.009; BMI > 30 kg/m^2^: RR 1.49; 95% CI 1.12–1.99; *p* = 0.006). There was no increased risk for EAL in patients with a BMI below vs. above 18 kg/m^2^ (RR 0.93; 95% CI 0.59–1.47; *p* = 0.74) (Fig. 2).

All studies were screened for information on frequency of alcohol consumption and the occurrence of EAL. Eight studies with 4216 patients [28, 36, 42, 52, 56, 74, 88] showed that patients with frequent alcohol intake had an increased risk of suffering from postoperative EAL with a RR 1.7 compared to patients without frequent alcohol intake (RR 1.7; 95% CI 1.2–2.41; *p* = 0.003) (Fig. 2).

To estimate the impact of smoking on EAL, meta-analysis of patients with current vs. no tobacco use (n = 17 studies/16,296 patients) [10, 28, 39, 42, 47, 49, 51, 55, 56, 71, 72, 74, 88–91] and of patients with former vs. no tobacco use (*n* = 7 studies/3892 patients) [3, 39, 42, 47, 57, 90] were performed. In patients with current (RR 1.25; 95% CI 1.13–1.38; *p* < 0.0001) and former tobacco use (RR 1.54; 95% CI 1.29–1.84; *p* < 0.0001), a significant higher risk of EAL compared to patients without tobacco use in their PMH could be shown (Fig. 2).

### Impact of Cardiovascular Risk Factors on the Development of EAL

**Fig. 3:**
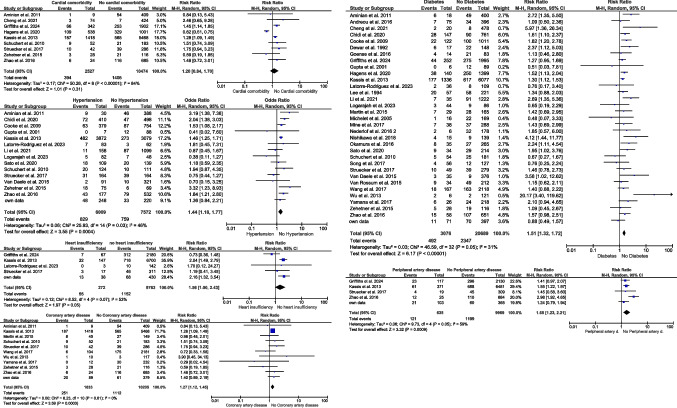
Impact of cardiovascular risk factors on the development of EAL

For cardiovascular comorbidities (hypertension, coronary artery disease, heart insufficiency), nine studies [1, 8, 10, 28, 37, 47, 64, 68, 91] with 13,001 patients were included in the meta-analysis. The overall estimated RR of 1.20 was higher for patients with cardiovascular comorbidities to exhibit an EAL after surgery compared to patients without cardiovascular comorbidities (RR 1.2; 95% CI 0.84–1.7; *p* = 0.31) (Fig. 3). Detailed analysis revealed an increased amount of EAL in patients with arterial hypertension [2, 8, 10, 28, 38, 39, 52, 55, 63, 64, 68, 71, 91, 92] compared to those without arterial hypertension (RR 1.44; 95% CI 1.18–1.77; *p* = 0.0004) (Fig. 3). Accordingly, coronary artery disease [8, 10, 28, 56, 64, 68, 73, 74, 88, 91] also revealed a noticeable increase of EAL (RR 1.26; 95% CI 1.11–1.43; *p* = 0.0004) (Fig. 3) as well as the presence of heart insufficiency [10, 47, 52, 68] (RR 1.56; 95% CI 1.00–2.43; *p* = 0.05) than in their controls (Fig. 3).

The RR of patients with (*n* = 3076) or without diabetes mellitus (*n* = 20,689) [1, 2, 8, 10, 28, 35, 37–39, 47, 52, 55–60, 63, 64, 67, 68, 71–74, 88, 89, 91–95] also revealed a significant increased risk of patients suffering from diabetes mellitus to develop EAL after esophagectomy compared to patients without diabetes mellitus (RR 1.51; 95% CI 1.32–1.72; *p* < 0.00001) (Fig. 3).

For patients with or without peripheral artery disease, a total of five studies [10, 28, 47, 68] including 10,604 patients were identified. In the presence of peripheral artery disease, the risk of EAL was significant higher with a RR of 1.65 compared to patients without peripheral artery disease (RR 1.65; 95% CI 1.23–2.21; *p* = 0.0009) (Fig. 3).

### Impact of Pulmonary and Renal Comorbidities on the Development of EAL

**Fig. 4:**
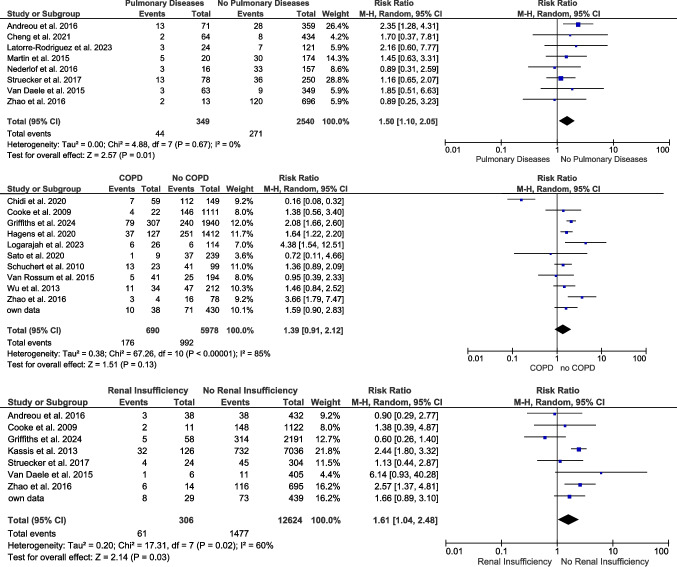
Impact of pulmonary and renal comorbidities on the development of EAL

A history of pulmonary disease (eight studies/2799 patients) [28, 35, 37, 52, 56, 68, 71, 96] and especially COPD (11 studies/6668 patients) [1, 28, 38, 39, 47, 55, 63, 64, 72, 73] were extracted and pooled in separate meta-analyses. The prevalence of pulmonary diseases (RR 1.5; 95% CI 1.1–2.05; *p* = 0.01) and of COPD (RR 1.39; 95% CI 0.91–2.12; *p* = 0.13) increased the risk of EAL (Fig. 4).

For renal insufficiency, an overall pooled RR of our meta-analysis including 12,930 patients of eight studies [10, 28, 35, 39, 47, 68, 71] showed a higher risk of developing EAL compared to patients without renal insufficiency (RR 1.61; 95% CI 1.04–2.48; *p* = 0.03) (Fig. 4).

### Impact of Cancer Specification on the Development of EAL

**Fig. 5:**
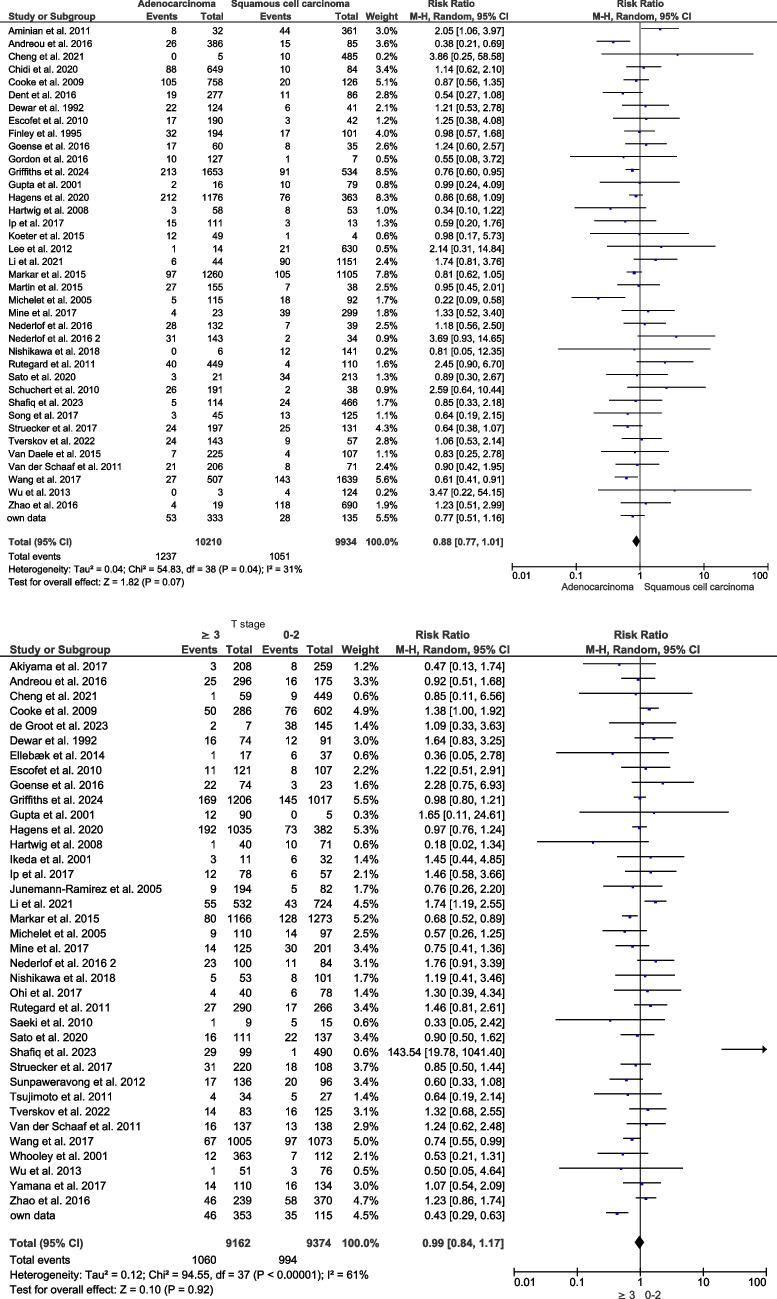
Impact of cancer specification on the development of EAL

No major differences were observed in the comparison of 39 studies including 20,144 patients [1, 2, 4, 5, 8, 26, 28, 35, 37–40, 44, 46, 47, 49, 53, 56–58, 60, 63–65, 67, 68, 70, 71, 73, 75, 77, 88, 89, 92, 93, 96] with adenocarcinoma of the esophagus versus squamous cell carcinoma (RR 0.88; 95% CI 0.77–1.01; *p* = 0.07) (Fig. 5).

Thirty-eight studies including 18,536 patients [1–5, 24, 26, 28, 34, 35, 37, 39, 41–43, 47, 49, 50, 58, 60–63, 65, 68–70, 73–76, 88, 89, 92, 93, 96] showed an equal distribution of low-risk (T < 3, 50.6%) and high-risk esophageal cancer (T ≥ 3, 49.4%). The meta-analysis showed no difference (RR 0.99; 95% CI 0.84–1.17; *p* = 0.92) for development of EAL depending on tumour stage (Fig. 5).

### Impact of Neoadjuvant Treatment on the Development of EAL

**Fig. 6:**
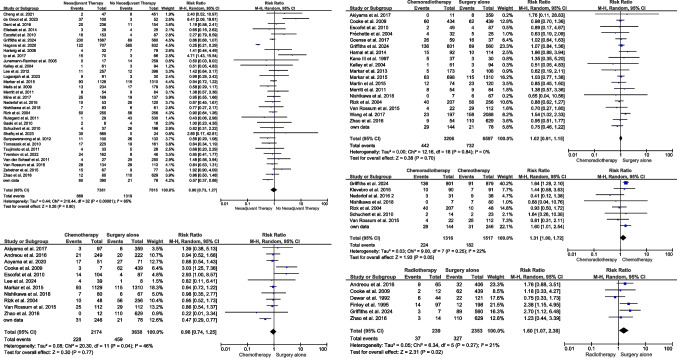
Impact of neoadjuvant treatment on the development of EAL

To assess the impact of oncological treatment and possible treatment of risk factors prior to surgery, comparative meta-analyses were conducted for upfront surgery vs. neoadjuvant treatment including nearly 15,000 patients form 33 studies [1, 4, 5, 24, 26, 28, 37, 40–43, 47, 49, 50, 53, 55, 58, 60, 62, 64–66, 69, 70, 72, 75, 86, 91, 96–99]. However, no effect was evident in the overall pooled RR of the meta-analysis with a risk ratio of 0.96 (95% CI 0.73–1.27; *p* = 0.8) (Fig. 6). To gain detailed insight into neoadjuvant treatment regimes, studies were stratified into upfront surgery vs. neoadjuvant chemotherapy (CTx) [5, 28, 34–36, 39, 43, 54, 60, 72, 99] or neoadjuvant radiochemotherapy (RCTx) [5, 28, 34, 39, 43, 47, 60, 72, 88, 89, 97–103] or CTx vs. RCTx [47, 60, 64, 72, 96, 99, 104] and in upfront surgery vs. radiotherapy (RTx) alone [28, 35, 39, 44, 47, 93]. However, neither surgery vs. neoadjuvant CTx (RR 0.96; 95% CI 0.74–1.25; *p* = 0.77) nor surgery vs. neoadjuvant RCTx (RR 1.02; 95% CI 0.91–1.15; *p* = 0.7) revealed any significant findings. Adding radiation protocols to neoadjuvant therapy could show a higher risk of developing EAL (RCTx vs. CTx (RR 1.31; 95% CI 1.00–1.72; *p* = 0.05), RTx alone vs. upfront surgery (RR 1.6; 95% CI 1.07–2.38; *p* = 0.02)) (Fig. 6).

### Analysis of Publication Bias

**Fig. 7:**
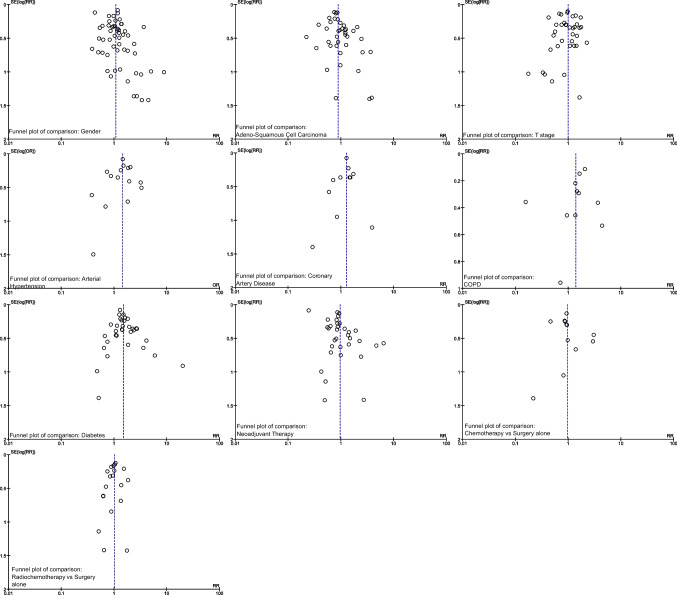
Analysis of publication bias

Cochrane handbook for systematic reviews advises to perform funnel plots of each meta-analysis containing at least ten studies. The symmetrical distribution of the funnel plots implies that no serious publication bias was present in these meta-analyses (Fig. 7).

### Impact of Prehabilitation

The idea of prehabilitation is not new and was first introduced in 2002 by Topp et al. [105]. However, studies on the impact of prehabilitation on the development of EAL after esophagectomy are still lacking. Modifiable factors that were identified by the meta-analyses are cardiovascular, pulmonary, renal, and dietary parameters. At our department, prehabilitational screening is initiated at the first contact regardless of the necessity of neoadjuvant therapy prior to surgery or not. The prehabilitation program emphasizes dietary interventions tailored to address obesity, malnutrition, or diabetes mellitus, alongside addiction counseling for patients with nicotine or alcohol use. Furthermore, patients are recommended to see cardiology, nephrology, or pulmonology to improve if existent their corresponding PMH. Since the efficacy of recommendation is heavily dependent on patient compliance, this analysis focuses on the outcomes related to prehabilitational screening.

The analysis revealed a reduction in the relative risk for EAL development in patients with a BMI > 30 kg/m^2^ (RR reduced from 1.49 to 1.12) and in those with a BMI > 25 kg/m^2^ (RR reduced from 1.31 to 1.3). Similarly, the relative risk for EAL was reduced in patients with diabetes mellitus (RR reduced from 1.51 to 0.88) and arterial hypertension (RR reduced from 1.44 to 1.36). In contrast, patients undergoing addiction counseling showed no significant reduction in EAL risk, with RR values for smokers remaining at 1.24 (from 1.25) and for individuals with alcohol use disorder at 1.77 (from 1.7). Nevertheless, patients benefited objectively from the prevention of perioperative withdrawal symptoms, underscoring the secondary advantages of addiction management.

## Discussion

EAL is one of the most serious and most devastating complications after esophagectomy increasing mortality rates up to 35% and accounting for approximately up to 50% of postoperative deaths [1, 75, 88]. Early detection and treatment of EAL is a crucial cornerstone to reduce the number of postoperative deaths caused by EAL after esophagectomy [43]. Several markers like serum c-reactive protein (CRP) [34, 101], serum procalcitonin C [101], and amylase of the chest tube [76] have already been described as potentially powerful for early prediction of EAL after esophagectomy. Current research focus on adjustment of neoadjuvant therapies as well as improvement of surgical techniques and procedures that do not show to positively affect postoperative EAL.

In this context, our current systematic review with meta-analysis which consisted in total of 51,396 esophagectomies with 5865 EAL provides a broad spectrum of patient-related risk factors. Highly significant markers for developing EAL after esophagectomy are the presence of regular alcohol intake (RR 1.7), peripheral artery disease (RR 1.65), renal insufficiency (RR 1.61), heart insufficiency (RR 1.56), diabetes mellitus (RR 1.51), pulmonary diseases (RR 1.5), a BMI > 30 kg/m^2^ (RR 1.49), and arterial hypertension (RR 1.44). Comparing the impact of cardiovascular, renal, and metabolic comorbidities to our in-house data, the effect on EAL risk appears significantly mitigated due to the consistent management of these factors prior to surgery. This underscores the potential benefit of structured prehabilitation programs in reducing modifiable risks, such as obesity, diabetes mellitus, and arterial hypertension, before esophagectomy. However, the limited impact of addiction counseling on EAL rates suggests that certain interventions may provide more indirect perioperative benefits rather than directly influencing surgical outcomes. Future randomized controlled trials are necessary to validate these findings and further elucidate the role of prehabilitation in optimizing patient outcomes following esophagectomy.

The meta-analysis for the impact of patients’ age on EAL showed no significant findings, whereas our data propose that patients with EAL are in average 2.67 years older than their controls but not the age to have a direct impact on EAL. Data on age dependent EAL in favor [106] and against [12, 98] is however quite old, mainly not in the focus of published studies and furthermore not influenceable prior to surgery.

Although the huge number of included patients and thus high level on information, limitations of this review with meta-analysis were caused by including all operation techniques, mainly retrospective data and no uniform definition or detection mode of EAL. Nevertheless, a broad spectrum of risk factors could be identified to increase the risk of EAL after esophagectomy.

## Conclusion

This comprehensive analysis underscores the multifactored nature of EAL as a significant postoperative complication following esophagectomy. Despite advancements in surgical techniques, patient outcomes remain heavily influenced by a combination of comorbidities and perioperative management. Our findings reaffirm that a high BMI, smoking, alcohol consumption, cardiovascular comorbidities, and other risk factors significantly increase the likelihood of EAL, while cancer specifics and surgical techniques have minor impact. These insights emphasize the necessity of tailored preoperative risk stratification and targeted interventions, such as prehabilitation programs, to mitigate modifiable risks.

By adopting a more holistic approach than the given risk assessment scores for postoperative complications and mortality that would integrate risk assessment, personalized care, and advanced treatment protocols, it would be feasible to not only lower complications but also enhance long-term effects like the quality of life and survival rates for patients undergoing esophagectomy.

## Data Availability

The entire systematic review and meta-analysis protocol was registered at PROSPERO (CRD42017060841). Center's data is provided within the manuscript and supplementary information files.
